# The Gathering Storm: Is Untreatable Typhoid Fever on the Way?

**DOI:** 10.1128/mBio.00482-18

**Published:** 2018-03-20

**Authors:** Myron M. Levine, Raphael Simon

**Affiliations:** a Center for Vaccine Development, University of Maryland School of Medicine, Baltimore, Maryland, USA

**Keywords:** ceftriaxone resistance, chloramphenicol, typhoid fever

## Abstract

Klemm et al. (mBio 9:e00105-18, 2018, https://doi.org/10.1128/mBio.00105-18) present comprehensive antibiotic sensitivity patterns and genomic sequence data on Salmonella enterica serovar Typhi blood culture isolates from typhoid fever cases during an epidemic in Pakistan. Microbiologic and genomic data pinpoint the identities and locations of the antimicrobial resistance genes and the outbreak strain’s lineage. They propose that Salmonella enterica serovar Typhi be added to the list of bacterial pathogens of public health importance that have become extensively drug resistant (XDR). This paper portends possible dire scenarios for typhoid fever control if XDR strains disseminate globally. Since the outbreak strain is of the H58 haplotype, known for its ability to spread worldwide and displace endemic *S*. Typhi, this concern is well-founded. The report of Klemm et al. forewarns the global community to address control of typhoid fever more aggressively through prevention, should therapeutic options disappear. This Commentary frames the Klemm et al. findings within a historic perspective.

## COMMENTARY

Most clinicians and clinical microbiologists today do not appreciate the lethality that typhoid fever exhibited in the preantibiotic era, when this acute generalized infection of the gut-associated lymphoid tissue, reticuloendothelial system, and gallbladder caused by Salmonella enterica serovar Typhi manifested a case fatality rate of ~15%. Typhoid fever was so common and lethal in the first decades of the 20th century in the United States that it constituted a rite of passage for school age children and young adults. *JAMA* published yearly updates and 5-year summaries that monitored the typhoid death toll in the most populous cities of the United States (1). The situation changed abruptly after 1948, when Theodore Woodward of the University of Maryland School of Medicine evaluated the efficacy of the antibiotic chloramphenicol to treat scrub typhus on the Malay Peninsula. He discovered that cases of typhoid fever that had unknowingly been enrolled into the study responded well to chloramphenicol therapy (2). This momentous discovery revolutionized the prognosis of typhoid fever, as the case fatality dropped to <1%, and it ushered in the antibiotic era for treating typhoid and paratyphoid fever. The ensuing 7 decades have chronicled an epic thrust-and-parry duel between the appearance of powerful new antibiotics and a wily bacterial foe’s stepwise acquisition of resistance to them. The report by Klemm et al. (3) in this issue of an epidemic of typhoid caused by an extensively drug-resistant (XDR) *S*. Typhi strain augurs the possibility that in the near future the practical and economically feasible options for treating typhoid in developing countries will be severely limited or even disappear, resulting in a return to the pre-1948 era when typhoid was not a treatable disease and mortality was high.

For the next 25 years following the hallmark 1948 discovery, chloramphenicol became used ubiquitously for the treatment of typhoid fever. Chloramphenicol was not an ideal drug, since a few percent of treated typhoid patients progressed to become chronic gallbladder carriers, relapses (milder than the initial illness) occurred in ~5 to 15% of treated cases, and fatal aplastic anemia was a rare severe adverse drug reaction (~1 in 24,500 to 40,800 patients). Nevertheless, prompt therapy with chloramphenicol led to abatement of fever, with a return to the person’s normal baseline within 4 to 5 days, and severe typhoid complications (e.g., intestinal perforation and hemorrhage) became uncommon. Most importantly, by preventing mortality, this oral antibiotic became the main tool for typhoid fever control in developing countries. During this period, from the perspective of acquisition of antimicrobial resistance (AMR), *S*. Typhi behaved differently than nontyphoidal *Salmonella* and *Shigella*, pathogens that readily acquired R factor plasmids that encode resistance to chloramphenicol and other antibiotics. In contrast, prior to 1972, there were only a few isolated reports of *S*. Typhi strains resistant to chloramphenicol. One early explanation was that, other than incompatibility group H1 (IncH1) R factor plasmids, the plasmids experimentally transferred were not stably maintained within *S*. Typhi (4). This situation ended unexpectedly in the early 1970s, as *S*. Typhi containing stable IncH1 plasmids that encoded chloramphenicol resistance caused an extensive epidemic in Mexico (5) and were also isolated in Vietnam (6). Large epidemics of chloramphenicol-resistant typhoid followed 1 decade later in Peru and Chile. Infectious disease researchers demonstrated in clinical trials in the early 1970s that the oral antibiotics amoxicillin and trimethoprim-sulfamethoxazole (TMP-SMZ) could adequately replace chloramphenicol (7, 8).

In South and Southeast Asia during the late 1980s and early 1990s, *S*. Typhi that carried R factor plasmids encoding resistance to most of the first-line antimicrobials used to treat typhoid fever, including chloramphenicol, amoxicillin, ampicillin, and TMP-SMZ, appeared (9, 10). Fortunately, by that time, ciprofloxacin, ofloxacin, and other fluoroquinolones had become available and proved to be highly efficacious anti-*S*. Typhi drugs. Not only did they achieve high clinical and bacteriologic cure rates, but few patients treated with fluoroquinolones became chronic gallbladder carriers and relapses were uncommon; indeed, a 4-week regimen of fluoroquinolone was ~90% efficacious in curing chronic typhoid gallbladder carriers, without cholecystectomy (11). However, promiscuous use of fluoroquinolones, often administered in suboptimal doses and for insufficient duration in Asia, encouraged the appearance of mutations in chromosomal *gyr* loci, resulting in resistance to fluoroquinolones. As multidrug-resistant (MDR) *S*. Typhi strains spread in the first decade of the millennium, several cephalosporin antibiotics, particularly parenteral ceftriaxone, became critical therapeutic tools.

Occasional reports of typhoid fever patients infected with ceftriaxone-resistant *S*. Typhi were published, but neither large outbreaks nor widespread endemic prevalence ensued. It is against this background that the paper by Klemm et al. is momentous (3). The authors report a sizable epidemic of typhoid fever in Sindh, Pakistan, caused by an *S*. Typhi strain carrying an array of both plasmid-borne and chromosome-borne resistance genes that collectively confer resistance to the main first-line oral antimicrobials used to treat typhoid, as well as to parenteral ceftriaxone. The only remaining reliable oral first-line antibiotic available is azithromycin.

The Sindh strain carries a composite transposon that is integrated into the chromosome at the *yidA* locus and encodes multiple resistances to chloramphenicol (*catA1*), amoxicillin-ampicillin (*bla*_TEM-1_), and TMP-SMZ (*dfrA7*, *sul1*, *sul2*), and it carries a single mutation in chromosomal *gyrA* (S83F), as found in many other H58 strains currently circulating in South Asia, that confers intermediate resistance to ciprofloxacin. In addition, the Sindh H58 clone carries an IncY plasmid, designated P60006 by Klemm et al. (3), that includes the plasmid quinolone resistance gene *qnrS* and the *bla*_CTX-M-15_ gene, which encodes an extended-spectrum β-lactamase conferring resistance to ceftriaxone; moreover, P60006 harbors a VirB Tra locus that allows self-transmission of this plasmid to other *S*. Typhi and *S*. Paratyphi A lineages.

The collective result of the panoply of chromosomal and plasmid AMR genes is that the Sindh outbreak *S*. Typhi encodes resistance to all the main antimicrobials that have been considered first-line drugs to treat typhoid fever during the past 70 years, including chloramphenicol, amoxicillin, ampicillin, TMP-SMZ, ciprofloxacin and other fluoroquinolones (e.g., ofloxacin), and ceftriaxone, and according to Klemm et al., it should be considered an XDR *S*. Typhi strain (12). For most of Pakistan’s health care system, the only remaining oral antibiotic available with proven efficacy is azithromycin. Strains resistant to azithromycin have already been reported in South Asia, and it is possible that increasingly widespread use of this azilide will accelerate the appearance of resistance to this antibiotic, as well (13, 14). The theoretical fallback antibiotics would then be expensive parenterally administered antimicrobials, such as carbapenems (imipenem-cilastatin combination, meropenem, ertapenem) and tigecycline (a glycylcycline). While these drugs represent options for treating patients in industrialized countries, their cost would be prohibitive for routine use as first-line drugs to treat typhoid fever cases in developing countries.

One may ponder what the likelihood is that this Andromeda-like XDR *S*. Typhi strain may spread widely beyond Pakistan? This is likely, as Klemm et al. have shown that the Sindh outbreak strain is of the dominant H58 haplotype, which exhibits a propensity to spread geographically and replace endemic clones once it invades a region (15). One traveler has already imported the Sindh strain into the United Kingdom (3).

The Pakistan outbreak caused by an XDR H58 strain of *S*. Typhi should be regarded as a clarion call that notifies public health authorities globally that we are rapidly approaching a scenario where the acquisition of one more resistance (to azithromycin) might result in an *S*. Typhi pathogen that is, in practical terms, virtually untreatable in most developing countries. This would return developing countries where typhoid is endemic to a pre-1948 scenario in which ~15% of cases of typhoid fever end in fatality.

We know how to impede amplified transmission of typhoid in most areas of endemicity, i.e., to treat water supplies and make them widely available and to improve sanitation and personal hygiene so that human feces do not contaminate water and food. While improvements in water supply and sanitation in developing countries must be the preferred long-term intervention to interrupt typhoid transmission, they are expensive and require time to deploy, even if political will and financing are available. In the interim, widespread use of typhoid vaccines can diminish the typhoid burden by modifying the susceptibility of populations at risk. A Vi conjugate vaccine was recently prequalified by the World Health Organization (WHO) and recommended for implementation in developing-country pediatric populations with high typhoid burdens (16); the GAVI Alliance has committed financing assistance for eligible countries to procure the Vi conjugate. Ty21a, a live oral vaccine, is also available to play a role.

Some may contend that with less-promiscuous antimicrobial usage in the community and enhanced stewardship in health care settings, the XDR strain will not disseminate widely and that sensitive *S*. Typhi will return. In parts of Asia, this has happened, as the prevalence of MDR strains has diminished and chloramphenicol-sensitive strains have reappeared. However, the contrary scenario is also feasible, i.e., that the Sindh XDR strain will disseminate in Asia, migrate to sub-Saharan Africa and perhaps Oceania, and infect unvaccinated travelers from industrialized countries. Moreover, the P60006 XDR plasmid may be transferred to *S*. Paratyphi A, as has already happened with MDR plasmids (17). In some South Asian populations, *S*. Paratyphi A comprises ~30 to 50% of enteric fever blood culture isolates. While there are live oral and conjugate vaccines to prevent *S*. Paratyphi A disease in clinical development, none are licensed. The global estimates of annual typhoid cases range from ~10.0 to 20 million, accompanied by ~130,000 to 210,000 deaths. Additionally, it is estimated that there are ~2 to 5 million cases and perhaps 20,000 to 40,000 deaths from paratyphoid A disease. Were a notable proportion of the enteric fever cases in the future to become virtually untreatable because they are caused by emergent H58 haplotype XDR strains, this would turn the clock back to the pre-1948 era, when typhoid fever was not treatable. Now is the time for global action to prevent a “gathering storm” from becoming a “perfect storm” and an enormous public health crisis.
